# The Tsushima leopard cat exhibits extremely low genetic diversity compared with the Korean Amur leopard cat: Implications for conservation

**DOI:** 10.7717/peerj.7297

**Published:** 2019-07-15

**Authors:** Hideyuki Ito, Miho Inoue-Murayama

**Affiliations:** 1 Kyoto City Zoo, Kyoto, Kyoto, Japan; 2 Wildlife Research Center, Kyoto University, Kyoto, Kyoto, Japan; 3 Wildlife Genome Collaborative Research Group, National Institute for Environmental Studies, Tsukuba, Japan

**Keywords:** Molecular genetic analysis, Tsushima leopard cat, Genetic diversity

## Abstract

We examined genetic diversity of the wild Tsushima leopard cat—a regional population of the Amur leopard cat—using microsatellite markers. In addition, we compared genetic diversity of the Tsushima leopard cat with that of the Korean population of Amur leopard cat. Although bias should be considered when applying cross-species amplification, the Tsushima leopard cat showed a lower index of molecular genetic diversity than did the Korean population. These results were consistent with those obtained using other genetic markers, such as mitochondrial DNA and Y chromosome sequences. This low genetic diversity of the wild Tsushima leopard cat may be derived from the founding population. Furthermore, our results suggest that the captive populations held in Japanese zoos may show extremely low genetic diversity, leading to difficulties in genetic management of the Tsushima leopard cat. Moreover, the two regional populations were clearly separated using these marker sets. In the present study, we demonstrated that the genetic diversity of the Tsushima leopard cat is extremely low compared with that of the continental regional population. Importantly, the Japanese captive population for *ex situ* conservation was derived from a founding population with extremely low genetic diversity; hence, we assume that both the captive and wild populations showed extremely low genetic diversities. Our findings emphasize the need to develop carefully considered management strategies for genetic conservation.

## Introduction

**Figure 1:**
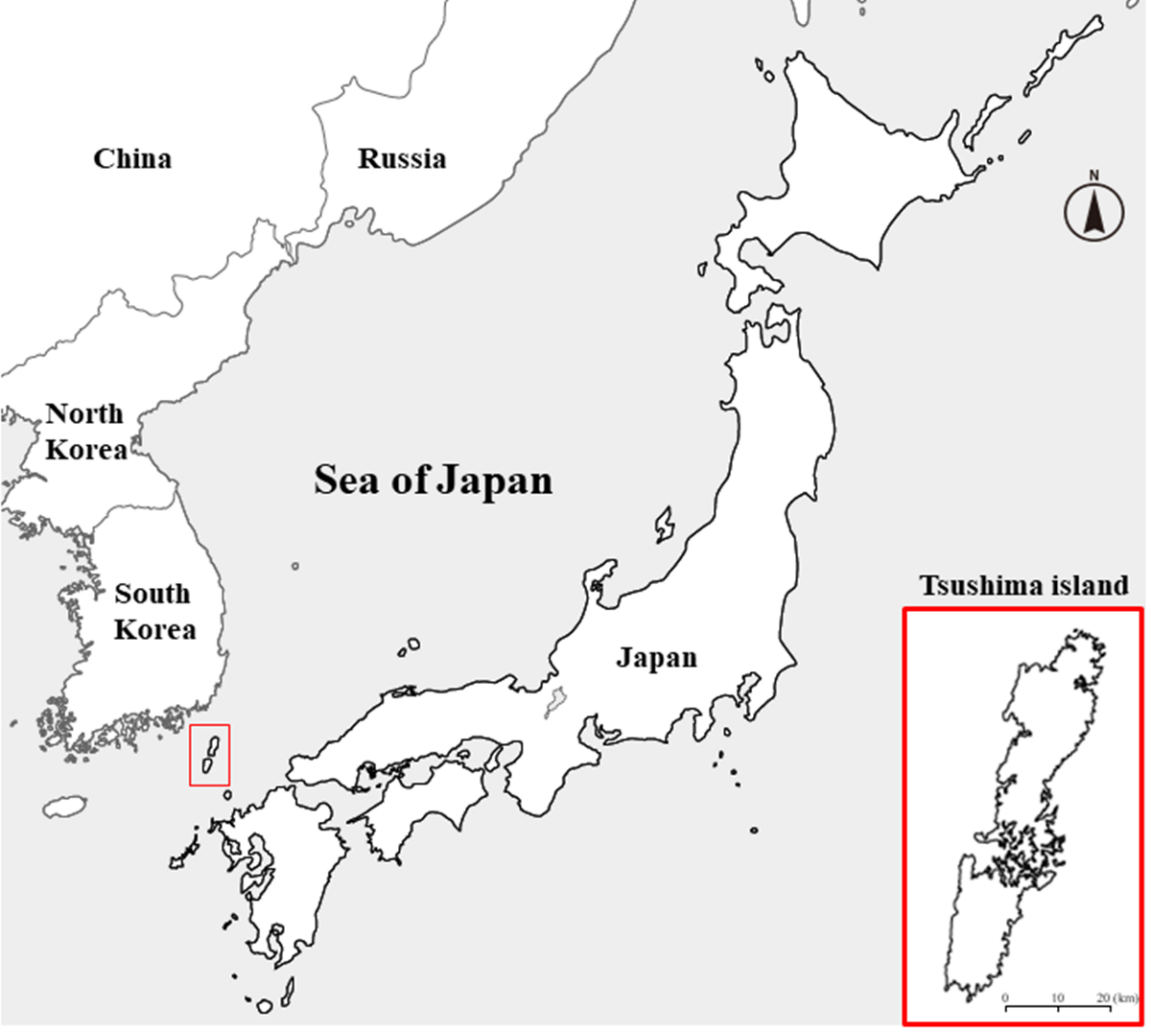
Location of Tsushima Island. This map shows the location and magnified view of Tsushima in East Asia.

The Tsushima leopard cat (*Prionailurus bengalensis euptilurus*) is a wild feline, which is restricted to the Tsushima Island in Japan (Fig. 1). The Tsushima leopard cat is a regional population of the Amur leopard cat (*P. bengalensis euptilurus*)—a subspecies of the leopard cat (*P. bengalensis*). The wild population of the Tsushima leopard cat has been declining, mainly because of habitat fragmentation, prey base depletion (rodents), and road kills. Their estimated number in the wild is less than 100 (Izawa et al., 2009). Therefore, the Tsushima leopard cat has been assigned the status of a National Nature Monument and has been classified as a critically endangered species in the Japanese Red List. In 1995, the Japanese Ministry of the Environment established the Protection and Breeding Program for the Tsushima Leopard Cat. The main objectives of this program were to confirm the habitat status, to maintain and improve the environment, to breed individuals in captivity, and to reintroduce individuals into the wild. Currently, the Tsushima leopard cats are kept in a limited number of zoos (nine) in Japan to minimize the risk of extinction, and the conservation project by the Japanese Association of Zoo and Aquarium (JAZA) for the Tsushima leopard cat promotes reproduction, maintains the captive population, and conducts research for conservation of this species. The JAZA conservation project was certified by the Japanese Ministry of the Environment as part of the National Protection and Breeding Program.

Detailed information and various findings related to nutrition, reproduction, husbandry, veterinary medicine, and genetics are required for the conservation of endangered species. In particular, genetic information is critical for small populations because such populations are more susceptible to various genetic effects, such as genetic drift, deleterious trait accumulation, and genetic polymorphism reduction. The population of the Tsushima leopard cat is very small; hence, a conservation strategy based on genetic information is essential. To date, some genetic research (involving mitochondrial DNA, Y-chromosomal DNA sequences, and microsatellites) has been conducted, and the results of such investigations have indicated that the Tsushima leopard cat shows lower genetic variation than do other populations of the Asian leopard cat (Masuda & Yoshida, 1995; Tamada et al., 2008). Microsatellites are one of the most frequently used nuclear markers for molecular ecology because of their many advantages, such as high polymorphism, codominant inheritance, ubiquitous abundance, and rapid mutation rates (Ellegren, 2004; Guichoux et al., 2011; Schlotterer, 2000). However, their application for research on endangered species with little existing genetic information is complicated by the laborious and time-consuming nature of work and high development costs. Recently, the use of next-generation sequencing techniques for the identification of microsatellite markers has been found to reduce both time and costs, and these techniques can serve as a powerful tool for genetic studies in non-model and endangered species. Eo et al. (2016) have identified 12 new microsatellite markers based on genomic information of the Korean Amur leopard cat and have tested these markers in several related species. Using molecular genetics, the divergence between the Tsushima and continental populations was estimated to have occurred approximately 100,000 years ago (Masuda & Yoshida, 1995). Because the Tsushima and Korean regional populations were geographically separated by a sea, there had been little gene flow after the separation of the two populations. Therefore, analysis and comparison of the two regional populations are essential for understanding the genetic structure and characteristics of the Tsushima leopard cat.

In the present study we aimed to assess the diversity of Tsushima leopard cat populations. We analyzed genetic polymorphism at 12 microsatellite loci identified in the population of the Korean Amur leopard cat and compared genetic variation indices between the Tsushima and Korean populations. Moreover, we evaluated genetic differentiation between the two populations using structure analysis and principal coordinate analysis (PCoA).

## Materials and Methods

### Sample collection

This study was conducted in strict accordance with the guidelines for the ethics in animal research established by the Wildlife Research Center of Kyoto University. Kyoto City Zoo approved the research (#20170426). We obtained muscle samples from 28 Tsushima leopard cats, which had been killed in road accidents or rescued during 2004–2012**.** All muscle samples were obtained from animals that died in road accidents or after the death of rescued individuals and were stored in ethanol until DNA extraction. DNA was extracted from the samples using the QIAGEN DNeasy Blood and Tissue Kit (Qiagen, Hilden, Germany).

### Microsatellite genotyping

Primers were re-designed to amplify repeated regions according to the reference sequences (Eo et al., 2016) using Primer 3, with a product length of 90–220 bp for non-invasive samples (Table 1). These microsatellite markers were synthesized with fluorescent labels and PCR reactions were performed by multi-plex and single-plex PCR (Table 1). The M13 universal forward sequence tag (M13F: 5′-GTTGTAAAACGACGGCCAGT-3′) or the Tail C sequence tag (Tail C: 5′-CAGGACCAGGCTACCGTG-3′) (Blacket et al., 2012) was added to the 5′ end of the forward primer of each pair to allow fluorescent labeling with 5-hexachloro-fluorescein or 6-carboxy-fluoresceine, respectively. PCR amplifications were performed using the modified protocol of the Qiagen Multiplex PCR Kit (Qiagen) in a final volume of 10 µl, containing 20 ng DNA, 5 µl Multiplex PCR Master Mix, 400 µM of each dNTP, 0.04 µM of each forward primers, 0.08 µM of each reverse primers, and 0.08 µM of M13 or Tail C primer (fluorescent labeled). PCR involved initial denaturation at 95 °C for 15 min, followed by 20 cycles of denaturation at 94 °C for 30 s, annealing at 57 °C for 30 s, and extension at 72 °C for 1 min; this was followed by 20 cycles of denaturation at 94 °C for 30 s, annealing at 52 °C (for M13F) or 55 °C (for Tail C) for 30 s, extension at 72 °C for 30 s, and final extension at 60 °C for 30 min. Amplicon size was assessed using the ABI PRISM 3130xl Genetic Analyzer (Applied Biosystems, Foster City, CA, USA). Genotypes were visually scored using GENEMAPPER (Applied Biosystems). PCR and genotyping were repeated more than three times depending on the genotype observed, to control for allelic drop-out.

**Table 1:** Primers of 12 microsatellite loci.

Locus	Labeled primer	Panel of multiplex PCR	Forward primer (5′-3′)	Reverse primer (5′-3′)	Repeat motif	GenBank
*Pbe01_M*	Tail C	1	tggagtttggcccgttcatt	acatgtctcagaaccgcaga	(AAAC)^5^	KU290367^a^
*Pbe02_M*	M13F	1	tggaaaagctcatttctttccact	gttgcactttgtaactttagttctaac	(AAGA)^5^	KU290368^a^
*Pbe03_M*	M13F	2	ctatgacccacactgccagg	ccctcaagatgcttaatatcagct	(ATAG)^6^	KU290369^a^
*Pbe05_M*	M13F	2	gcagggactcagtaaatgtttagc	ccggttggatctcatctgga	(TGAA)^5^	KU290371^a^
*Pbe06_M*	Tail C	2	ggaaaacttgaggactgccc	ggctctttgatgcccactttc	(AAAT)^9^	KU290372^a^
*Pbe09_M*	Tail C	2	agtgaaagttgctggactctga	acaagggaacattacaaccact	(AACA)^5^	KU290375^a^
*Pbe10_M*	M13F	3	gagctttgagcagcaatggg	tggccagccacaatattaca	(AAGG)^12^	KU290376^a^
*Pbe13_M*	Tail C	3	gcaaatctgcggatgttggg	caggccgagaccagttaagg	(GGAA)^11^	KU290379^a^
*Pbe15_M*	M13F	3	cagtcagcatcgatcatgacc	gcaaataaggtttggatattggtgc	(TAAA)^10^	KU290381^a^
*Pbe26_M*	Tail C	3	acccacacttgtgtctctgc	cctgcagttacaatcaaactcgt	(AATG)^8^	KU290387^a^
*Pbe31_M*	M13F	1	gcttgctaatgtcaggggtt	gcttcagatgcagatttgggt	(GATA)^5^	KU290392^a^
*Pbe32_M*	M13F	1	gcttttcaggtttcacgatatgc	tctctctgttctttctggggc	(GTTT)^5^	KU290393^a^

**Notes.**

Sequences of M13F and Tail C are 5′-GTTGTAAAACGACGGCCAGT-3′ and 5′-CAGGACCAGGCTACCGTG-3′, respectively. Reverse primers are labeled at the 5′ end with pigtail sequences (5′-GTTTCTT-3′).

a Genbank accession number from Eo et al. (2016).

To compare the genetic structure and diversity of the Tsushima population with other regional populations, data from a previous dataset from the Korean population were used (*n* = 30) (Eo et al., 2016). The coincidence between the genotypes reported in the previous study (Eo et al., 2016) and that observed in this study was confirmed by sequencing PCR products at 12 loci in two Tsushima leopard cats and comparing the number of repetitions of microsatellite motifs between the two studies. Alleles with the same number of repetitions of microsatellite motifs in both studies were assumed to be the same. PCR products were purified using the High Pure PCR Purification Kit (Roche, Switzerland), sequenced using the Big Dye Terminator ver.3.1 Cycle Sequencing Kit (Applied Biosystems) with PCR primers (forward and reverse) based on the manufacturer’s protocol, and electrophoresed using an ABI PRISM 3130×l sequencer. Forward and reverse complement sequences were aligned to obtain a consensus sequence using MEGA ver. 6.0 (Tamura et al., 2013).

### Genetic diversity analysis

Allelic richness (*Ar*) per locus was calculated using the HP-Rare software ver. 1.1 (Kalinowski, 2005). The number of alleles (*Na*) and private alleles (*Np*), expected heterozygosity (*He*) and observed heterozygosity (*Ho*), probability of identity (*Pid*), and *Pid* among siblings (*Pid-sib*) were calculated using GenALEx ver. 6.5 (Peakall & Smouse, 2012). Deviation from the Hardy–Weinberg equilibrium and linkage disequilibrium were tested using GENEPOP ver. 4.0.10 (Rousset, 2008) after Bonferroni correction. Effective population size was calculated using NeEstimator ver. 2 (Do et al., 2014). In the Korean population, three variables (*Na*, *He* and *Ho*) were cited data from the previous study (Eo et al., 2016), while the other variables were calculated as part of this study. *F*_*IS*_ (population differentiation between individuals among sampled populations), *F*_*IT*_ (population differentiation among individuals), and *F*_*ST*_ (population differentiation between populations) were calculated using GENEPOP ver. 4.0.10 (Rousset, 2008).

### Genetic structure analysis

Microsatellite data were analyzed using the program STRUCTURE ver. 2.3.4 (Pritchard, Stephens & Donnelly, 2000). For this analysis, an admixture model was employed to estimate the genetic structure of the population and individual ancestries both among and within populations. Using Markov chain Monte Carlo methods, we conducted the analysis with 10 iterations for each population size (*K*) of 1–10 that were run for 500,000 generations after an initial burn-in of 250,000 generations. The most probable number of clusters (*K*) was determined by calculating the second order rate of change considering the likelihood of *K* between successive *K* values (Evanno, Regnaut & Goudet, 2005) using STRUCTURE HARVESTER (Earl & vonHoldt, 2012). Runs were averaged using CLUMPP ver. 1. 1. 2 (Jakobsson & Rosenberg, 2007), and the results were visualized using Structure Plot (Ramasamy et al., 2014). Moreover, the pattern of allelic differentiation between species was assessed using PCoA with GenALEx ver. 6.5 (Peakall & Smouse, 2012) based on the calculated genetic distances.

## Results

### Genetic diversity

Genetic diversity indices of the population of the Tsushima leopard cat are summarized in Table 2. All loci were successfully amplified, and only four loci were polymorphic. Moreover, 12 loci ×2 population combinations showed no deviations from the Hardy–Weinberg equilibrium after sequential Bonferroni correction. Cumulative *Pid* values for all loci in the Tsushima and Korean populations were 3. 6 × 10^−6^ and 2. 1 × 10^−1^, respectively. Cumulative *Pid-sib* values for all loci in the Tsushima and Korean populations were 3. 5 × 10^−3^ and 4. 5 × 10^−1^, respectively. Effective population sizes (95% CI) of the Tsushima and Korean populations were 8.2 (0.8–infinity) and 115.1 (33.3–infinity), respectively.

**Table 2:** Genetic diversity indices of two regional populations of the Amur leopard cat with 12 microsatellite markers.

Locus	Korea^a^									Japan								
	*N*	*Na*	*Np*	*Ne*	*Ho*	*He*	*Ar*	*Pid*	*Pid-sib*	*N*	*Na*	*Np*	*Ne*	*Ho*	*He*	*Ar*	*Pid*	*Pid-sib*
*Pbe01_M*	30	2	1	1.301	0.267	0.231	2.000	0.618	0.789	28	1	0	1.000	0.000	0.000	1.000	1.000	1.000
*Pbe02_M*	30	3	2	1.835	0.467	0.455	2.933	0.386	0.619	28	1	0	1.000	0.000	0.000	1.000	1.000	1.000
*Pbe03_M*	30	5	3	2.532	0.667	0.605	4.993	0.214	0.501	28	2	0	2.000	0.714	0.500	2.000	0.375	0.594
*Pbe05_M*	30	2	1	1.142	0.133	0.124	2.000	0.774	0.881	28	1	0	1.000	0.000	0.000	1.000	1.000	1.000
*Pbe06_M*	29	3	2	2.393	0.690	0.582	3.000	0.240	0.519	28	1	0	1.000	0.000	0.000	1.000	1.000	1.000
*Pbe09_M*	30	2	0	1.260	0.233	0.206	2.000	0.652	0.810	28	3	1	1.244	0.214	0.196	3.000	0.656	0.816
*Pbe10_M*	30	7	6	4.327	0.867	0.769	6.996	0.085	0.387	28	1	0	1.000	0.000	0.000	1.000	1.000	1.000
*Pbe13_M*	30	4	2	2.359	0.500	0.576	3.933	0.255	0.526	28	2	0	1.036	0.036	0.035	2.000	0.932	0.965
*Pbe15_M*	30	5	4	2.442	0.533	0.591	4.933	0.205	0.506	28	1	0	1.000	0.000	0.000	1.000	1.000	1.000
*Pbe26_M*	30	2	1	1.514	0.300	0.339	2.000	0.494	0.704	28	2	1	1.036	0.036	0.035	2.000	0.932	0.965
*Pbe31_M*	30	4	3	1.410	0.300	0.291	3.930	0.524	0.736	28	1	0	1.000	0.000	0.000	1.000	1.000	1.000
*Pbe32_M*	30	3	2	1.448	0.300	0.309	2.997	0.508	0.722	28	1	0	1.000	0.000	0.000	1.000	1.000	1.000
Average		3.50	2.25	1.997	0.438	0.423	3.476	0.413	0.642		1.42	0.07	1.110	0.083	0.064	1.417	0.908	0.945

**Notes.**

*N* number of individuals *Na* number of alleles *Np* number of private alleles *Ne* the number of effective alleles *Ho* observed heterozygosity *He* expected heterozygosity *Ar* allelic richness *Pid* probability of identity *Pid* − *sib* *Pid* among siblings

a In Korean population, three variables (*Na*, *He* and *Ho*) were cited data from the previous study (Eo et al., 2016), while the other variables were calculated as part of this study.

### Differentiation of the two regional populations

**Figure 2:**
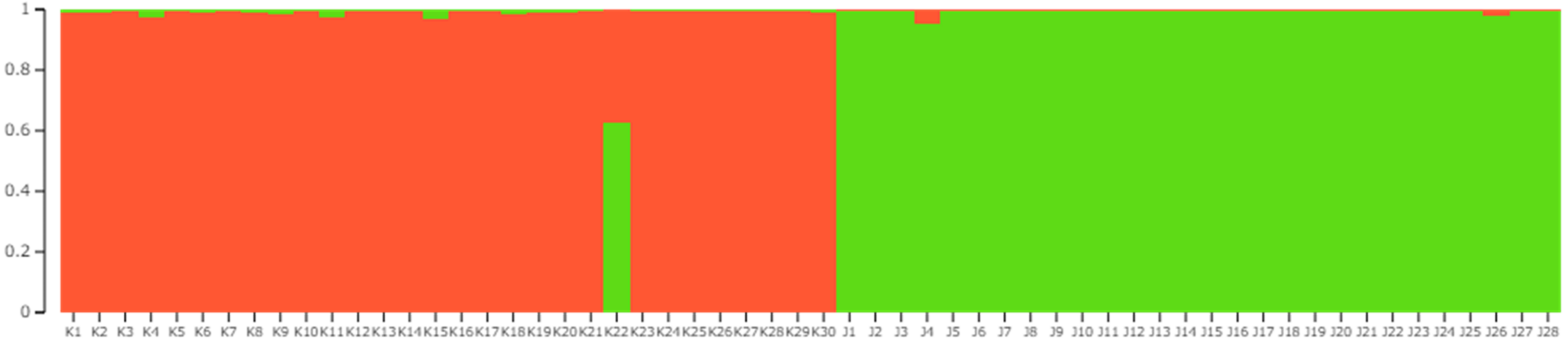
Bayesian analysis of the genetic structure showing the differentiation of the two regional populations of the Amur leopard cat based on 12 microsatellite loci. This figure was obtained using Structure Plot and CLUMPP to align the 10 replicates for optimal *K* = 2 (all runs were performed using the Markov chain Monte Carlo method running for 500,000 generations and an initial burn-in of 250,000 generations). K1-K30: Korean population. J1-J28: Tsushima leopard cat.

**Figure 3:**
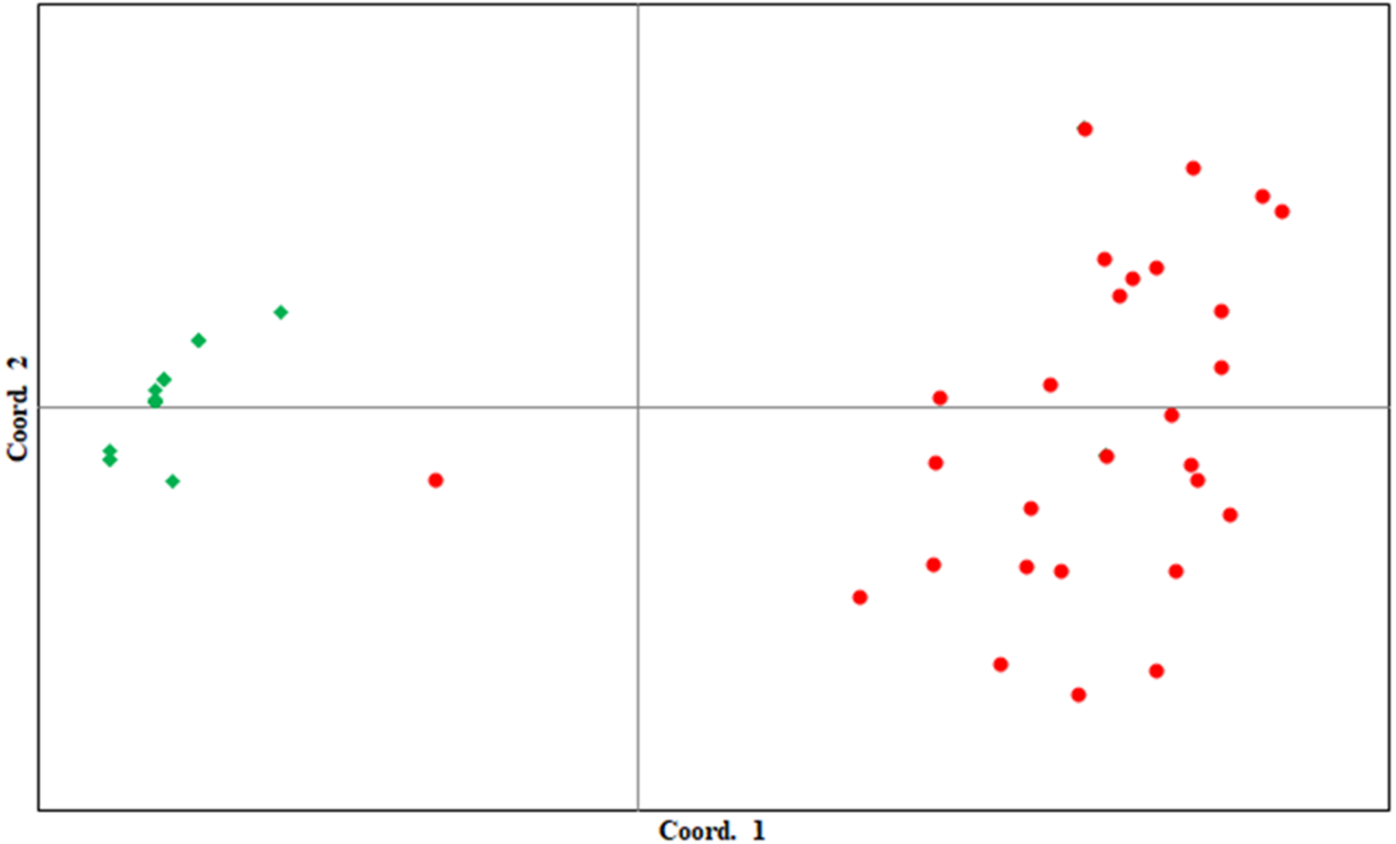
First and second components of principal coordinate analysis of 12 microsatellite loci in the two regional populations of the Amur leopard cat. Percentages of variation explained by the first two axes were 53.27% and 7.36%, respectively. Diamond symbols: Tsushima leopard cat. Circle symbols: Korean population.

*F*_*IS*_, *F*_*ST*_, and *F*_*IT*_ between the Tsushima and Korean populations across all loci were −0.0504, 0.4803, and 0.4541, respectively. Clustering of the two populations observed in the STRUCTURE (Fig. 2) and principal component analyses (Fig. 3) demonstrates a clear separation of the two populations using microsatellite data, except for one individual. In the STRUCTURE analysis, the greatest support was found for *K* = 2 clusters, resulting in the separation of the Tsushima leopard cat and the Korean leopard cat populations (Fig. 2). In addition, the two populations were clearly separated in PCoA, except for one individual. The percentages of variation explained by the first two axes were 53.27% and 7.36%, respectively (Fig. 3).

## Discussion

### Diversity of the Tsushima leopard cat

The Tsushima population showed lower molecular genetic diversity, as represented by number of alleles, heterozygosity, and allelic richness, than did the Korean regional population (Table 2). This pattern was consistent with the results of previous research on mitochondrial DNA and the Y chromosome (Masuda & Yoshida, 1995; Tamada et al., 2008). Because DNA markers, such as SNPs and microsatellites, were developed to optimize analysis in a specific species for which the markers were developed, bias should be considered when these markers are applied to other species (Brandstrom & Ellegren, 2008). Because the present study involved the comparison between the two regional populations that belong to the same subspecies, the bias is considered to be smaller than the bias observed in comparison between different species. In addition, microsatellite markers have little ascertainment bias compared with SNPs (Li et al., 2008). Therefore, the profound difference between the two populations indicates that the genetic diversity of the Tsushima population is extremely low compared with that of the Korean population. To reduce the effect of bias due to marker development, genome-wide analyses, such as double-digest restriction site-associated DNA sequencing (Zhou et al., 2014) and multiplexed ISSR genotyping by sequencing (Suyama & Matsuki, 2015), are warranted. Because genome-wide analyses can simultaneously conduct genotyping at massive polymorphic sites, the effect of bias can be reduced and genetic diversity can be evaluated more accurately. Moreover, this approach can help resolve the genetic relationship between the two populations and further clarify the evolution and divergence of the leopard cat.

Cumulative *Pid* and *Pid-sib* values were high in both the regional populations, particularly in the Tsushima population. These *Pid-sib* values showed that the developed markers were unable to identify all individuals in both the populations. Therefore, more microsatellite markers should be developed to identify individuals, which can help clarify the ecology of leopard cats. The *F*_*IS*_ value was high because of a high *F*_*ST*_ value; this result indicates that the two regional populations are differentiated, but the total population has a high inbreeding coefficient. Effective population size of the Tsushima leopard cat was very low, indicating that the Tsushima leopard cat may be affected by the bottleneck effect and/or genetic drift. However, the calculated effective population size had a wide range (upper limit was infinity); therefore, more individuals and loci should be analyzed to establish a more precise estimate of population size.

### Genetic structure

STRUCTURE analysis and PCoA using the developed markers clearly distinguished between the two regional populations, except for one individual. These results indicate that the two populations are genetically separated. The leopard cat has been classified into four subspecies based on recent molecular genetic studies, and the Tsushima leopard cat is classified as the Amur leopard cat similar to the Korean population. The Amur leopard cats have no subdivision among their habitat (Patel et al., 2017). In our study, the two populations were clearly separated; however, further analyses are required since only a very small part of the genome was analyzed. In addition, the percentage of variation explained by axis 1 in PCoA was very high (53.27%), indicating that a single locus had a large contribution to the genetic segregation of the populations. There were two private alleles in the Tsushima population and 27 in the Korean population. The Korean population had many private alleles and appeared to show high genetic diversity. However, the Korean Amur leopard cat population has been reported to have low genetic diversity compared with other feline and endangered species (Ko et al., 2018). Therefore, the relatively high number of private alleles observed in the Korean population may be due to comparison with the Tsushima leopard cat, which has very low genetic diversity. The observed genotype tended to shift with genotyping on different sequencers and scoring by different researchers. Therefore, to reduce genotyping error between the two studies, it is important to genotype a subset of samples used in the study by Eo et al. (2016) together with samples of the Tsushima leopard cats and to confirm the genotyping between the two studies. The divergence time between the Tsushima and continental populations has been estimated to be 100,000 years ago (Masuda & Yoshida, 1995). Considering their geographical distribution, the divergence of the Korean and Tsushima populations was estimated to have occurred at around the same time. Therefore, the lower genetic diversity and smaller number of private alleles in the Tsushima population were probably derived from a founder effect when the Tsushima population diverged from the continental populations. We cannot judge whether Tsushima leopard cats were affected by a bottleneck effect after divergence because of the small number of analyzed samples and polymorphic loci (four). Thus, identification and analysis of more microsatellite loci (minimum number of polymorphic loci >20) are imperative to understand the genetic background of the Tsushima leopard cat (Luikart et al., 1998). The loci that differ greatly in allele frequency between the Tsushima and Korean populations are speculated to have been derived through the founder effect. Specific alleles in the Tsushima leopard cat may have been acquired through mutations after divergence from the continental population or from individuals carrying the alleles as a minor allele type in the continental population that arrived at the Tsushima Island as founders. Although the analysis of 25–30 individuals is sufficient to evaluate the genetic diversity of a population (Hale, Burg & Steeves, 2012), when comparing regional populations considered to have experienced a founder effect, the analysis of more individuals is essential to investigate the origins of specific alleles. In this survey, despite the separation between the two populations, one individual in the Korean population exhibited genetic types from both the populations (Tsushima and Korea). There may be several reasons for this occurrence. First, some regions in Korea may be genetically separated from other regions, retaining the genetic type of both the populations. Second, there may be gene flow from Tsushima to Korea. Finally, this may be a simple typing error caused by the use of different devices. However, to reduce error derived from this problem, we sequenced two genotypes at each locus in this study and compared them with those from previous studies. In recent years, genome-wide analyses have been reported to clarify the extent of gene flow and divergence among populations (Prado-Martinez et al., 2013). Therefore, increasing the number of analyzed individuals and performing genome-wide analysis will help elucidate the histories of both the populations.

Collectively, these results provided insights into initiating a captive conservation breeding program for Tsushima leopard cats. For many endangered species, conservation breeding programs have been established with the primary goal of maintaining the genetic diversity of populations to prevent the negative effect of inbreeding and preserve the adaptive potential of the species (Frankham, Ballou & Briscoe, 2010). Traditionally, the goal of many breeding programs has been to retain 90% of the genetic diversity observed in the founding population for 100 years (Frankham, Ballou & Briscoe, 2010). However, because of the potential for relatedness among founders, the true level of genetic diversity in subsequent generations is likely to be lower than that estimated based on pedigree analysis. Previously, we have clarified the discordance between molecular genetic analysis and pedigree analysis in two zebra species: Grevy’s and Hartmann’s mountain zebras (Ito et al., 2017). The combination of molecular genetics and traditional pedigree analyses has recently been employed in several captive breeding programs to resolve these issues (Ferrie et al., 2013; Gautschi et al., 2003; Henkel et al., 2012; Ivy et al., 2009; McGreevy Jr, Dabek & Husband, 2011; Ogden et al., 2007). Such combined analyses can allow us to evaluate genetic diversity based on both analyses and to thereby develop more appropriate genetic management strategies. The captive population of Tsushima leopard cat has 21 founders that introduced to captive population from wild in 1996–2015. However, only eight individuals have made a genetic contribution to the current population. This study indicates that the wild population of Tsushima leopard cats has a very low genetic diversity; hence, the founders of the captive population were highly likely to be genetically closely related and to have low genetic diversity. This fact indicates that the breeding program for Tsushima leopard cats should be carefully managed. Although genetic evaluations for all founders have been the same in the traditional management strategy based on pedigrees, molecular genetic analysis can indicate high-priority individuals for breeding.

The present study showed that the Tsushima leopard cats have an extremely low genetic diversity compared with the Korean population, and this marker set could distinguish between the two regional populations. However, in the present study, we analyzed only a small fraction of the genome, which does not necessarily provide an accurate estimate of individual genetic diversity. Genome-wide data are already used for research and conservation of endangered species, such as gorillas (Xue et al., 2015) and Visayan warty pigs (Bosse et al., 2015). Using these advanced applications, establishment of a more effective breeding plan and clarification of precise genetic diversity are possible in the future. Therefore, performing a genome-wide analysis of the Tsushima leopard cats is warranted.

## Conclusions

1. The Tsushima leopard cat population has a lower genetic diversity in nuclear DNA, as evidenced by molecular genetic analysis, than does the regional Korean continental population, which belongs to the same subspecies: the Amur leopard cat.

2. STRUCTURE and PCoA analyses revealed that the two regional populations are clearly divided.

3. Our findings suggest that captive populations may have extremely low genetic diversity, and better planned genetic management is required for the conservation of the Tsushima leopard cats.

##  Supplemental Information

10.7717/peerj.7297/supp-1 Table S1 Genotypes of 28 Tsushima leopard cat in 12 microsatellite loci These data were obtained from row typing score of multiple genotyping experiments.

## Additional Information and Declarations

### Data Availability

The following information was supplied regarding data availability: The raw measurements are available in File S1.

## References

[ref-1] Blacket MJ, Robin C, Good RT, Lee SF, Miller AD (2012). Universal primers for fluorescent labelling of PCR fragments—an efficient and cost-effective approach to genotyping by fluorescence. Molecular Ecology Resources.

[ref-2] Bosse M, Megens HJ, Madsen O, Crooijmans RP, Ryder OA, Austerlitz F, Groenen MA, De Cara MA (2015). Using genome-wide measures of coancestry to maintain diversity and fitness in endangered and domestic pig populations. Genome Research.

[ref-3] Brandstrom M, Ellegren H (2008). Genome-wide analysis of microsatellite polymorphism in chicken circumventing the ascertainment bias. Genome Research.

[ref-4] Do C, Waples RS, Peel D, Macbeth GM, Tillett BJ, Ovenden JR (2014). NeEstimator v2: re-implementation of software for the estimation of contemporary effective population size (Ne) from genetic data. Molecular Ecology Resources.

[ref-5] Earl D, vonHoldt B (2012). STRUCTURE HARVESTER: a website and program for visualizing STRUCTURE output and implementing the Evanno method. Conservation Genetics Resources.

[ref-6] Ellegren H (2004). Microsatellites: simple sequences with complex evolution. Nature Reviews Genetics.

[ref-7] Eo SH, Ko BJ, Lee B-J, Seomun H, Kim S, Kim M-J, Kim JH, An J (2016). A set of microsatellite markers for population genetics of leopard cat (*Prionailurus bengalensis*) and cross-species amplification in other felids. Biochemical Systematics and Ecology.

[ref-8] Evanno G, Regnaut S, Goudet J (2005). Detecting the number of clusters of individuals using the software STRUCTURE: a simulation study. Molecular Ecology.

[ref-9] Ferrie GM, Cohen OR, Schutz P, Leighty KA, Plasse C, Bettinger TL, Hoffman EA (2013). Identifying parentage using molecular markers: improving accuracy of studbook records for a captive flock of marabou storks (Leptoptilos crumeniferus). Zoo Biology.

[ref-10] Frankham R, Ballou JD, Briscoe DA (2010). Introduction to conservation genetics.

[ref-11] Gautschi B, Muller JP, Schmid B, Shykoff JA (2003). Effective number of breeders and maintenance of genetic diversity in the captive bearded vulture population. Heredity.

[ref-12] Guichoux E, Lagache L, Wagner S, Chaumeil P, LÉGer P, Lepais O, Lepoittevin C, Malausa T, Revardel E, Salin F, Petit RJ (2011). Current trends in microsatellite genotyping. Molecular Ecology Resources.

[ref-13] Hale ML, Burg TM, Steeves TE (2012). Sampling for microsatellite-based population genetic studies: 25 to 30 individuals per population is enough to accurately estimate allele frequencies. PLOS ONE.

[ref-14] Henkel JR, Jones KL, Hereford SG, Savoie ML, Leibo SP, Howard JJ (2012). Integrating microsatellite and pedigree analyses to facilitate the captive management of the endangered Mississippi sandhill crane (Grus canadensis pulla). Zoo Biology.

[ref-15] Ito H, Ogden R, Langenhorst T, Inoue-Murayama M (2017). Contrasting results from molecular and pedigree-based population diversity measures in captive zebra highlight challenges facing genetic management of zoo populations. Zoo Biology.

[ref-16] Ivy JA, Miller A, Lacy RC, Dewoody JA (2009). Methods and prospects for using molecular data in captive breeding programs: an empirical example using parma wallabies (Macropus parma). Journal of Heredity.

[ref-17] Izawa M, Doi T, Nakanishi N, Teranishi A (2009). Ecology and conservation of two endangered subspecies of the leopard cat (Prionailurus bengalensis) on Japanese islands. Biological Conservation.

[ref-18] Jakobsson M, Rosenberg NA (2007). CLUMPP: a cluster matching and permutation program for dealing with label switching and multimodality in analysis of population structure. Bioinformatics.

[ref-19] Kalinowski ST (2005). hp-rare 1.0: a computer program for performing rarefaction on measures of allelic richness. Molecular Ecology Notes.

[ref-20] Ko BJ, An J, Seomun H, Lee MY, Eo SH (2018). Microsatellite DNA analysis reveals lower than expected genetic diversity in the threatened leopard cat (Prionailurus bengalensis) in South Korea. Genes Genomics.

[ref-21] Li JZ, Absher DM, Tang H, Southwick AM, Casto AM, Ramachandran S, Cann HM, Barsh GS, Feldman M, Cavalli-Sforza LL, Myers RM (2008). Worldwide human relationships inferred from genome-wide patterns of variation. Science.

[ref-22] Luikart G, Allendorf FW, Cornuet JM, Sherwin WB (1998). Distortion of allele frequency distributions provides a test for recent population bottlenecks. Journal of Heredity.

[ref-23] Masuda R, Yoshida MC (1995). Two Japanese wildcats, the Tsushima cat and the Iriomote cat, show the same mitochondrial DNA lineage as the leopard cat Felis bengalensis. Zoological Science.

[ref-24] McGreevy Jr TJ, Dabek L, Husband TP (2011). Genetic evaluation of the Association of Zoos and Aquariums Matschie’s tree kangaroo (Dendrolagus matschiei) captive breeding program. Zoo Biology.

[ref-25] Ogden R, Langenhorst T, McEwing R, Woodfine T (2007). Genetic markers and sample types for pedigree reconstruction in Grevy’s zebra (Equus grevyi). Der Zoologische Garten.

[ref-26] Patel RP, Wutke S, Lenz D, Mukherjee S, Ramakrishnan U, Veron G, Fickel J, Wilting A, Forster DW (2017). Genetic structure and phylogeography of the leopard cat (Prionailurus bengalensis) inferred from mitochondrial genomes. Journal of Heredity.

[ref-27] Peakall R, Smouse PE (2012). GenAlEx 6.5: genetic analysis in Excel. Population genetic software for teaching and research—an update. Bioinformatics.

[ref-28] Prado-Martinez J, Sudmant PH, Kidd JM, Li H, Kelley JL, Lorente-Galdos B, Veeramah KR, Woerner AE, O’Connor TD, Santpere G, Cagan A, Theunert C, Casals F, Laayouni H, Munch K, Hobolth A, Halager AE, Malig M, Hernandez-Rodriguez J, Hernando-Herraez I, Prufer K, Pybus M, Johnstone L, Lachmann M, Alkan C, Twigg D, Petit N, Baker C, Hormozdiari F, Fernandez-Callejo M, Dabad M, Wilson ML, Stevison L, Camprubi C, Carvalho T, Ruiz-Herrera A, Vives L, Mele M, Abello T, Kondova I, Bontrop RE, Pusey A, Lankester F, Kiyang JA, Bergl RA, Lonsdorf E, Myers S, Ventura M, Gagneux P, Comas D, Siegismund H, Blanc J, Agueda-Calpena L, Gut M, Fulton L, Tishkoff SA, Mullikin JC, Wilson RK, Gut IG, Gonder MK, Ryder OA, Hahn BH, Navarro A, Akey JM, Bertranpetit J, Reich D, Mailund T, Schierup MH, Hvilsom C, Andres AM, Wall JD, Bustamante CD, Hammer MF, Eichler EE, Marques-Bonet T (2013). Great ape genetic diversity and population history. Nature.

[ref-29] Pritchard JK, Stephens M, Donnelly P (2000). Inference of population structure using multilocus genotype data. Genetics.

[ref-30] Ramasamy RK, Ramasamy S, Bindroo BB, Naik VG (2014). STRUCTURE PLOT: a program for drawing elegant STRUCTURE bar plots in user friendly interface. Springerplus.

[ref-31] Rousset F (2008). genepop’007: a complete re-implementation of the genepop software for Windows and Linux. Molecular Ecology Resources.

[ref-32] Schlotterer C (2000). Evolutionary dynamics of microsatellite DNA. Chromosoma.

[ref-33] Suyama Y, Matsuki Y (2015). MIG-seq: an effective PCR-based method for genome-wide single-nucleotide polymorphism genotyping using the next-generation sequencing platform. Scientific Reports.

[ref-34] Tamada T, Siriaroonrat B, Subramaniam V, Hamachi M, Lin LK, Oshida T, Rerkamnuaychoke W, Masuda R (2008). Molecular diversity and phylogeography of the Asian leopard cat, Felis bengalensis, inferred from mitochondrial and Y-chromosomal DNA sequences. Zoological Science.

[ref-35] Tamura K, Stecher G, Peterson D, Filipski A, Kumar S (2013). MEGA6: molecular evolutionary genetics analysis version 6.0. Molecular Biology and Evolution.

[ref-36] Xue Y, Prado-Martinez J, Sudmant PH, Narasimhan V, Ayub Q, Szpak M, Frandsen P, Chen Y, Yngvadottir B, Cooper DN, De Manuel M, Hernandez-Rodriguez J, Lobon I, Siegismund HR, Pagani L, Quail MA, Hvilsom C, Mudakikwa A, Eichler EE, Cranfield MR, Marques-Bonet T, Tyler-Smith C, Scally A (2015). Mountain gorilla genomes reveal the impact of long-term population decline and inbreeding. Science.

[ref-37] Zhou X, Xia Y, Ren X, Chen Y, Huang L, Huang S, Liao B, Lei Y, Yan L, Jiang H (2014). Construction of a SNP-based genetic linkage map in cultivated peanut based on large scale marker development using next-generation double-digest restriction-site-associated DNA sequencing (ddRADseq). BMC Genomics.

